# Importance of geographic origin for invasion success: A case study of the North and Baltic Seas versus the Great Lakes–St. Lawrence River region

**DOI:** 10.1002/ece3.2528

**Published:** 2016-10-21

**Authors:** Isabel Casties, Hanno Seebens, Elizabeta Briski

**Affiliations:** ^1^ GEOMARHelmholtz‐Zentrum für Ozeanforschung KielKielGermany; ^2^ Senckenberg Biodiversity and Climate Research CentreFrankfurt am MainGermany

**Keywords:** Baltic Sea, biodiversity, Great Lakes–St. Lawrence River, nonindigenous species, North Sea, Ponto‐Caspian region

## Abstract

Recently, several studies indicated that species from the Ponto‐Caspian region may be evolutionarily predisposed to become nonindigenous species (NIS); however, origin of NIS established in different regions has rarely been compared to confirm these statements. More importantly, if species from certain area/s are proven to be better colonizers, management strategies to control transport vectors coming from those areas must be more stringent, as prevention of new introductions is a cheaper and more effective strategy than eradication or control of established NIS populations. To determine whether species evolved in certain areas have inherent advantages over other species in colonizing new habitats, we explored NIS established in the North and Baltic Seas and Great Lakes–St. Lawrence River regions—two areas intensively studied in concern to NIS, highly invaded by Ponto‐Caspian species and with different salinity patterns (marine vs. freshwater). We compared observed numbers of NIS in these two regions to expected numbers of NIS from major donor regions. The expected numbers were calculated based on the available species pool from donor regions, frequency of shipping transit, and an environmental match between donor and recipient regions. A total of 281 NIS established in the North and Baltic Seas and 188 in the Great Lakes–St. Lawrence River. Ponto‐Caspian taxa colonized both types of habitats, saltwater areas of the North and Baltic Seas and freshwater of the Great Lakes–St. Lawrence River, in much higher numbers than expected. Propagule pressure (i.e., number of introduced individuals or introduction effort) is of great importance for establishment success of NIS; however in our study, either shipping vector or environmental match between regions did not clarify the high numbers of Ponto‐Caspian taxa in our study areas. Although we cannot exclude the influence of other transport vectors, our findings suggest that the origin of the species plays an important role for the predisposition of successful invaders.

## 1 Introduction

Anthropogenic introductions of species to new areas increase due to globalization and climate change, leading to homogenization of biodiversity worldwide (Capinha, Essl, Seebens, Moser, & Pereira, 2015; Hellmann, Byers, Bierwagen, & Dukes, 2008; Hulme, 2009; Olden, Poff, Douglas, Douglas, & Fausch, 2004). Species are incidentally transported with commercial travel and trade, such as in ships’ ballast water, wood packing materials, and horticultural soils, or are intentionally introduced like for games or biocontrol (Briski et al., 2013; Hulme et al., 2008; Lockwood, Hoopes, & Marchetti, 2007). Many species fail to establish a viable population after arriving to a new environment, but those that succeed may have significant consequences for local communities, ecosystem functioning and/or services to human society (Carlton & Geller, 1993; Chapin et al., 2000; Olden et al., 2004; Simberloff et al., 2013). Though empirical and statistical evidence suggests that propagule pressure (i.e., number of introduced individuals) is of crucial importance for establishment success (Hayes & Barry, 2008; Simberloff, 2009), population characteristics such as phenotypic plasticity and preadaptation to cope with changeable environmental conditions may keep a high propagule pressure of species while passing through the stages of the invasion process (i.e., transport, introduction, establishment, and spread; Colautti & MacIsaac, 2004; Lande, 2015). Moreover, species evolved in regions known as more geologically and environmentally disturbed and challenged may possess life‐history traits, higher phenotypic plasticity, or adaptational and evolutionary capacity which would enable them to be more successful invaders (Reid & Orlova, 2002). If species from certain area/s are proven to be better colonizers, management strategies to control transport vectors coming from those areas must be more stringent, as prevention of new species introductions is a cheaper and more effective strategy than eradication or control of established NIS populations (Hulme et al., 2008; Lockwood et al., 2007; Lodge et al., 2006).

**Figure 1:**
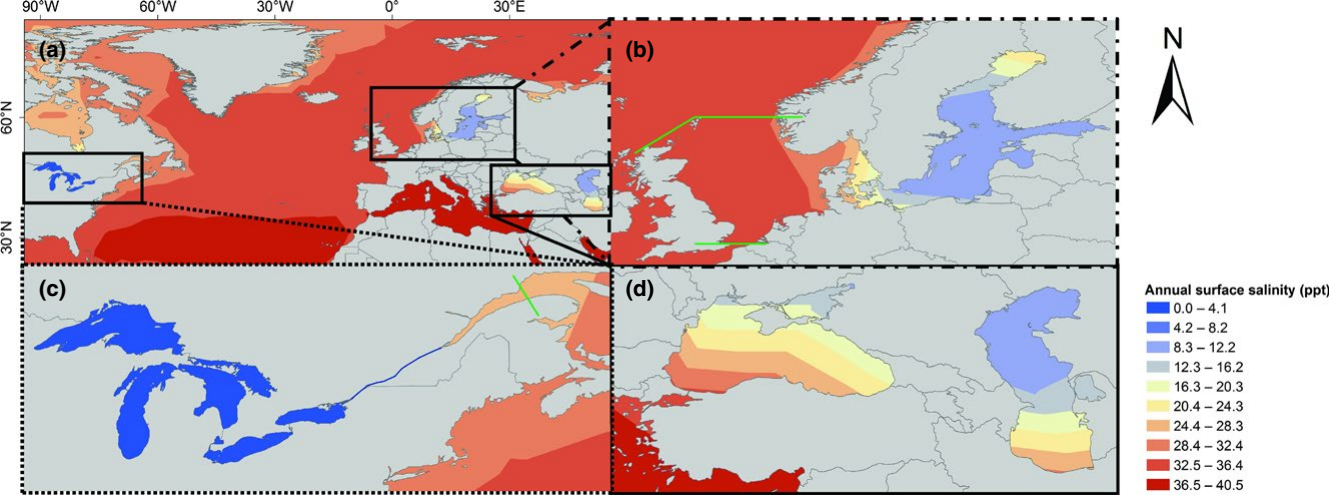
Salinity of the Great Lakes–St. Lawrence River, North Atlantic Ocean, North, Baltic, Mediterranean, Black, Azov, and Caspian Seas, constructed using average annual salinity data with a 1° x 1° spatial resolution from the World Ocean Atlas database (Antonov et al., 2006) (a). Close‐up maps of the North and Baltic Seas (b), the Great Lakes–St. Lawrence River (c), and the Black, Azov, and Caspian Seas (d) are shown, as well. The green lines mark the boundaries of the studied areas. Although, the salinity of the Great Lakes is shown in the range from 0.0 to 4.1 (i.e., dark blue), the salinity of the Great Lakes is under 0.5 ppt (i.e., freshwater)

After the opening of canals that link the North and Baltic Seas with the Black and Caspian Seas (the Rhine‐Main‐Danube, Volga‐Don and Volga‐Baltic Canals), species from the Ponto‐Caspian region (i.e., Black, Azov and Caspian Seas; Figure 1) spread and became abundant in freshwater and estuarine ports of northern Europe (Leppäkoski et al., 2002; Ricciardi & MacIsaac, 2000). The invasion history of the Laurentian Great Lakes tells a more intriguing story, with many Ponto‐Caspian species establishing in the region after invasion of Europe (Leppäkoski et al., 2002; Ricciardi & MacIsaac, 2000). Shipping is a leading mechanism for the spread of aquatic nonindigenous species (NIS) globally (Molnar, Gamboa, Revenga, & Spalding, 2008; Ricciardi, 2006), and as ship transit between the North and Baltic Seas and the Great Lakes–St. Lawrence River is relatively high and of similar intensity in both directions (Kaluza, Kölzsch, Gastner, & Blasius, 2010), one would expect a similar ratio of NIS from the Great Lakes–St. Lawrence River in the North and Baltic Seas and vice versa. However, recent studies stated that the transfer of species has been asymmetrical, with only a small number of species from the Great Lakes having invaded Northern European waters (Leppäkoski, Gollasch, & Olenin, 2010; Reid & Orlova, 2002).

The North and Baltic Seas and the Great Lakes–St. Lawrence River regions are intensively explored systems, and probably the most studied areas with regard to aquatic NIS globally (AquaNIS, 2015; DAISIE, 2015; Gollasch, Haydar, Minchin, Wolff, & Reise, 2009; Great Lakes Aquatic Nonindigenous Information System (GLANSIS) database, 2014; Pyŝek et al., 2008; Reise, Gollasch, & Wolff, 1999; Ricciardi, 2006). Both regions are geologically young water bodies formed by glaciations (Leppäkoski et al., 2002; Reid & Orlova, 2002). Their habitat types represent an interesting inverse mirror image with the North and Baltic Seas being mostly marine ecosystem with several large brackish to freshwater estuaries, while the Great Lakes–St. Lawrence River region is predominantly a freshwater environment with a huge brackish to saline St. Lawrence River estuary and Gulf of St. Lawrence (Figure 1; Antonov, Locarnini, Boyer, Mishonov, & Garcia, 2006; Environment Canada, 2013; Pocklington, 1986; Reid & Orlova, 2002). Both systems are marginal water bodies of the North Atlantic Ocean (Pocklington, 1986). Despite their opposing salinity patterns, some parts of the systems are rather alike: in particular, the Baltic Sea and the Lower St. Lawrence River. The Baltic Sea is a large semi‐enclosed brackish water area characterized by a strong salinity gradient ranging between 2 and 24 ppt (Leppäkoski et al., 2002), while the salinity of the St. Lawrence River, though freshwater in large part of the river stretch, starts to increase from Quebec City (5 ppt) reaching 24–32 ppt in the Gulf of St. Lawrence (Environment Canada, 2013; Pocklington, 1986). The climate in the Baltic Sea and St. Lawrence River is also similar, ranging from maritime temperate to continental subarctic climate (Pocklington, 1986).

Both, the North and Baltic Seas, and the Great Lakes–St. Lawrence River regions are heavily invaded by Ponto‐Caspian taxa, while at the same time, the systems are different in concern to salinity (marine vs. freshwater); in this study, we explored origin and taxonomic composition of NIS in these two regions to determine whether Ponto‐Caspian taxa have inherent advantages over other species in colonizing new areas. We compared observed numbers of NIS in these two regions (i.e., established) to expected numbers of NIS from major donor regions. The expected numbers of NIS were estimated based on the available species pool from donor regions, frequency of shipping transit, and an environmental match between donor and recipient regions. We tested the hypothesis that there is no difference between expected and observed numbers of NIS in the two regions. We also tested the hypotheses that there is no difference in (1) number of established NIS; (2) geographic origin of NIS; and (3) taxonomic composition of NIS between the two regions.

## 2 Material and Methods

### 2.1 Observed numbers of NIS, their origin, and taxonomic composition

Lists of aquatic NIS were compiled for the North and Baltic Seas, and the Great Lakes–St. Lawrence River region, respectively. The North and Baltic Seas NIS list (Appendix S1) was assembled using data from AquaNIS—the information system on aquatic nonindigenous and cryptogenic species (AquaNIS, 2015), Reise et al. (1999), Bij de Vaate, Jazdzewski, Ketelaars, Gollasch, and Van der Velde (2002), and Gollasch et al. (2009). The region was defined as the area affiliated to the Baltic Sea and North Sea and was confined by a line between Dover and the Belgian border and a line between the Shetland Islands to Norway (AquaNIS, 2015). The Great Lakes–St. Lawrence River's NIS alien species list (Appendix S2) was assembled from de Lafontaine and Costan (2002), Ricciardi (2006), and the Great Lakes Aquatic Nonindigenous Information System (GLANSIS) database (GLANSIS, 2014). The region was defined as the area of the Great Lakes basin and its ordinarily attached channels, wetlands and waters, and the St. Lawrence River until the Gulf of St. Lawrence River (Ricciardi, 2006; Great Lakes Aquatic Nonindigenous Information System (GLANSIS) database, 2014). The Gulf of St. Lawrence River was not included.

**Figure 2:**
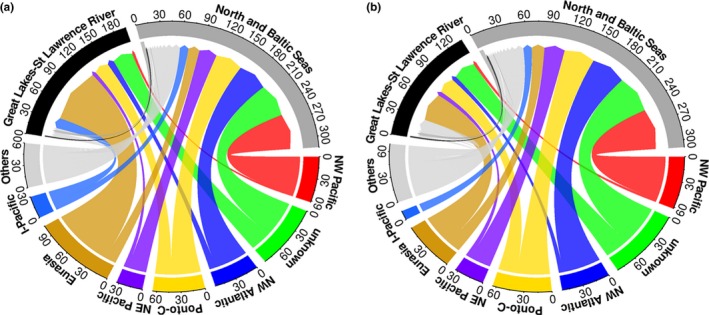
Flows of aquatic nonindigenous species from different regions to the North and Baltic Seas and the Great Lakes–St. Lawrence River region including Tracheophyta (a) and excluding Tracheophyta (b). The arrows at the end of the flows show toward the recipient region. If a species is native to two regions, it is shown as two flows in the plot. Each region is a color assigned and represented by a circle segment: northwest (NW) Pacific, unknown region, northwest (NW) Atlantic, Ponto‐Caspian region (Ponto‐C.), northeast (NE) Pacific, Eurasia (inland freshwaters except Yangtze River), North (N) America (inland freshwaters except the Laurentian Great Lakes, St. Lawrence, and Mississippi Rivers), Indo‐Pacific (I‐Pacific; Indian Ocean and the archipelago of Indonesia, Malaysia, and Pilipinas). Others include: northeast Atlantic, Mediterranean Sea, southeast Atlantic, southwest Pacific, Arctic, southwest Atlantic, Africa (inland freshwaters), Baltic Sea, New Zealand (inland freshwaters), North Sea, southeast Pacific, Yangtze River, Mississippi River, Australia (inland freshwaters), and South America (inland freshwaters)

To examine whether species from the Ponto‐Caspian region are more common NIS and whether an opposing salinity pattern of the two systems has an effect on established taxa, geographic origin of NIS and their taxonomic composition were determined. Geographic origin of species was assigned based on AquaNIS (2015) for the North and Baltic Seas NIS, while that of the Great Lakes–St. Lawrence River NIS was based on Great Lakes Aquatic Nonindigenous Information System (GLANSIS) database (2014). If information was not available on these two websites, a general internet search engine was conducted. Geographic origin was assigned to one or more groups: northeast Atlantic, northwest Atlantic, southeast Atlantic, southwest Atlantic, northeast Pacific, northwest Pacific, southeast Pacific, southwest Pacific, North Sea, Baltic Sea, the Great Lakes–St. Lawrence River region, Mediterranean Sea, Eurasia (inland freshwaters except Yangtze River), Mississippi River, Yangtze River, Arctic, Australia (inland freshwaters), New Zealand (inland freshwaters), Indo‐Pacific (Indian Ocean and the archipelago of Indonesia, Malaysia, and Pilipinas), Africa (inland freshwaters), North America (inland freshwaters except the Laurentian Great Lakes, St. Lawrence and Mississippi Rivers), South America (inland freshwaters), Ponto‐Caspian region and unknown region. If a species was native to two or more regions, its contribution was counted as a ratio of “one” over the number of regions that the species was native to. For example, if a species was native to two regions, the value of 0.5 has been assigned to each region. However in Figure 2, if a species was native to two or more regions, it was shown as two or more flows in the plot. Taxonomic assignments were based on several websites (e.g., Barcode of Life Database (BOLD), European Nature Information System (EUNIS), World Register of Marine Species (WORMS), and ZipcodeZoo). Species were assigned to kingdom, phylum, and class. Due to a high number of Tracheophyta species (vascular plants) established in the Great Lakes–St. Lawrence River region, and the fact that Tracheophyta is a mostly terrestrial and freshwater phylum with rare representatives in marine habitats (Bell & Hemsley, 2000; Les & Cleland, 1997), the results of our study are shown with and without this phylum.

### 2.2 Expected numbers of NIS

To calculate expected numbers of NIS from major donor regions for the North and Baltic Seas and Great Lakes–St. Lawrence River region, we first estimated average species richness for major donor regions using derived global species richness data from Tittensor et al. (2010). We calculated average species richness for a particular donor region by adding derived species richness of all coastal grids (880‐km resolution equal‐area grid) of that region, and then divided this total derived species richness with the number of coastal grids in that region. The average species richness of coastal grids was used to avoid overestimation of species richness due to a potential overlap of the same species from neighboring grids. Tittensor et al. (2010) data did not provide species richness for the Black and Caspian Seas nor for the Great Lakes–St. Lawrence River region; therefore, we calculated an average species richness for these regions using total species richness data from the European Environment Agency (EEA, 2016) and National Oceanic and Atmospheric Administration (NOAA, 2016). As the northwest Atlantic data were available in both, in Tittensor et al. (2010) as per 880‐km resolution equal‐area grids and in Marine Species Registers for the Northwest North Atlantic Ocean (MSRNNAO, 2016) as total species richness, we used these data to derive the correction factor. The correction factor was then applied to the total species richness data from the European Environment Agency (EEA, 2016) and National Oceanic and Atmospheric Administration (NOAA, 2016) to calculate average species richness for the Black and Caspian Seas and the Great Lakes–St. Lawrence River region compatible to the rest of our data (i.e., per 880‐km resolution equal‐area grids). The correction was necessary as total species richnesses per regions were approximately ten times higher than species richness per 880‐km resolution equal‐area grids.

In the second step, the obtained estimated average species richness was multiplied by the probability of invasion between regions to get the expected numbers of NIS transported from a donor to a recipient region. The expected number of NIS was used as a null model of NIS exchanges irrespective of species’ traits, which can be compared with the observed NIS exchanges. The invasion probabilities were calculated using the statistical model of Seebens, Gastner, and Blasius (2013). The model integrates global ship movement data, biogeographical similarity, and environmental conditions of ports worldwide to obtain the likelihood that a NIS is transported in ballast water from a donor port, released in a recipient port and able to establish a new population there.

According to Seebens et al. (2013), the model consists of three independent probabilities each denoting an important step of the invasion process: first, the ballast water released at site *j* may contain species from all regions previously entered by the ship including NIS but also species which are native to the recipient site *j*. This is accounted for by the probability to be nonindigenous

$$P_{ij}\left(\text{Nonindigenous}\right)={\left(1+\frac{\gamma }{d_{ij}}\right)}^{-\beta },$$

describing the probability that a species native at donor port *i* is nonindigenous in recipient port *j*. *P*_*ij*_(nonindigenous) is a sigmoidal function of geographic distance *d*_*ij*_ between the ports, with β and γ being constants, and can be interpreted as the proportion of NIS inoculated in the ballast water of a ship and transported over a certain distance.

Second, the probability of introduction describes the likelihood that a species is introduced from port *i* to port *j* on ship route *r*:

$$P_{r}\left(\text{Intro}\right)=\left(1-e^{-\lambda B_{r}}\right)e^{-\mu \Delta t_{r}}.$$

It increases with the amount of released ballast water *B*_*r*_ that originates from port *i* on ship route *r* and decreases with mortality rate μ and travel time Δ*t*_*r*_ between *i* and *j*. Ship routes were established from nearly 3 Mio. port calls (arrival and departure dates at ports) of 32,511 ships during 2007–2008. The arrival and departure dates as well as ship‐specific information were reported by the automatic identification system (AIS) and provided by Lloyd's Register Fairplay (www.ihs.com). For each port call of a ship, *B*_*r*_ was calculated depending on the ship type, ship size, the mean ballast water tank volume, and the past route of the ship. For a certain ship type and ship size, a mean volume of discharged ballast water was calculated from 717,250 ballast water release protocols provided by the National Ballast Information Clearinghouse for the USA (NBIC 2012). Although these data are restricted to the USA, they represent by far the most comprehensive collection of ballast water release protocols currently available. To estimate the amount of discharged ballast water originating from port *i*, we require the mean ballast water tank volumes for different size classes and ship types, which were obtained from the American Bureau of Shipping (ABS 2011). While assuming a constant release of ballast water at each port of call, we were then able to calculate for each port call of a ship the mean ballast water volume *B*_*r*_ originating from port *i* and discharged at port *j*. Travel times Δ*t*_*r*_ were extracted from these ship routes.

Third, the probability of establishment describes the likelihood that a species native at port *i* is able to establish a population in the recipient port *j*:

$$P_{ij}\left(\text{Estab}\right)=\alpha e^{-\frac{1}{2}\left[{\left(\frac{\Delta T_{ij}}{\sigma_{T}}\right)}^{2}+{\left(\frac{\Delta S_{ij}}{\sigma_{S}}\right)}^{2}\right]}$$

*P*_*ij*_(Estab) is a Gaussian function of differences in water temperatures *T* and salinities *S* normalized by standard deviations σ_*T*_ and σ_*S*_, and α being a constant.

The product of the three probabilities gives the probability of invasion *P*_*ij*_(Inv). To obtain invasion probabilities between regions, the invasion probabilities from all ports *a* in the donor region *A* to all ports  *b* in the recipient region *B* were aggregated according to *P*_*A,B*_(Inv) = 1–Π_a,b_(1–*P*_*a,b*_(Inv)). The parameter setting was adopted from Seebens et al. (2013), where more details of the underlying data, the model itself, model validation, and a sensitivity analysis can be found. The calculated invasion probabilities were multiplied with the estimated average species richness of a donor region to get the expected number of NIS from a particular donor region to a particular recipient region. Eurasia (inland freshwaters except Yangtze River) and North America (inland freshwaters except the Laurentian Great Lakes, St. Lawrence and Mississippi Rivers), two major donor regions for the Great Lakes–St. Lawrence River region, were excluded from this analysis due to a lack of shipping data. We emphasize here that our expected numbers of NIS from major donor regions to recipient regions could not be taken as absolute numbers of NIS in the recipient regions, but only as rough estimates due to the possibility that other vectors than shipping may operate between the regions and due to temporal changes in vector strength (e.g., number of arriving ships per time). A necessary time for species to have a chance to be transported and established in the recipient regions was also not taken into account in our calculations. Therefore, by considering only one transport vector, we underestimated expected numbers of NIS, but at the same time by not including time necessary for species to be transported and established, we overestimated those numbers. Finally, observed and expected numbers of NIS from each major donor region to each recipient region were statistically compared using chi‐square tests (performed in R version 3). Additional chi‐square tests were performed for each of the ten pairs of donor and recipient region separately.

Additionally, to better illustrate salinity patterns of the two systems and their connections to the Ponto‐Caspian region, we constructed a salinity map showing the Great Lakes–St. Lawrence River, North Atlantic Ocean, North, Baltic, Mediterranean, Black, Azov, and Caspian Seas (Figure 1). The map was constructed using average annual salinity data with a 1° x 1° spatial resolution from the World Ocean Atlas database of the National Oceanic and Atmospheric Administration (NOAA)'s National Oceanographic Data Centre (NODC) United States Department of Commerce (Antonov et al., 2006) by ArcGIS, ESRI Inc. All supplementary data of this study are available at https://doi.org/10.1594/PANGAEA.864713.

## 3 Results

### 3.1 Observed numbers of NIS, their origin, and taxonomic composition

A total of 281 NIS established in the North and Baltic Seas region, of which 156 established only in the North Sea and 53 only in the Baltic Sea; the establishment of 72 species overlap in the two water bodies (Table S1). In the Great Lakes–St. Lawrence River region, 188 NIS established with 104 only in the Great Lakes, three only in the St. Lawrence River, and 81 in both areas (Table S2). NIS occurring only in the St. Lawrence River were two fishes, *Oncorhynchus clarkii* and *Tinca tinca*, and one crustacean *Oronectes limosus* (Table S2).

Geographic origins of NIS differed between the two regions (Figure 2). While for 19% of NIS in the North and Baltic Seas region donor areas were unknown (54 species), the next dominant donors were the northwest Pacific (17%, 49 species), northwest Atlantic (16%, 44 species), and Ponto‐Caspian region (15%, 42 species; Figure 2, Tables 1 and S1). Contrary to the North and Baltic Seas, the most dominant donors for the Great Lakes–St. Lawrence River region were Eurasia (47%, 88 species) followed by 17% of species from unknown areas (31 species), 12% from the Ponto‐Caspian region (23 species), and nine percent from North America (17 species; Figure 2, Tables 1 and S2). After excluding Tracheophyta from the datasets, the most dominant donor regions did not change in either of the two regions (Figure 2). Yet, percentages of donors for the Great Lakes–St. Lawrence River region changed due to a high number of Tracheophyta NIS in that region (56 species; Table S2), which mostly originated from one region (86% from Eurasia); the new donor region percentages were 30, 23, 17, and 12% for Eurasia, unknown region, Ponto‐Caspian region, and North America, respectively (Figure 2). Twenty‐five species were recorded in both the North and Baltic Seas region and the Great Lakes–St. Lawrence River region (Tables S1 and S2). Eleven of these were from the Ponto‐Caspian region, four from both the northeast and northwest Pacific, while the rest originated from Eurasia, northwest Atlantic, North America, northeast Pacific, Yangtze River, New Zealand, or unknown region (Tables 1, S1 and S2).

**Table 1:** List of Ponto‐Caspian species established in the North and Baltic Seas, and the Great Lakes and the St. Lawrence River regions, and their taxonomic assignment

Taxon	Species	North Sea	Baltic Sea	Great Lakes	St. Lawrence River
Animalia					
Annelida					
Clitellata	*Paranais frici*		x		
	*Potamothrix bedoti*		x	x	
	*Potamothrix heuscheri*		x		
	*Potamothrix moldaviensis*			x	
	*Potamothrix vejdovskyi*		x	x	
Polychaeta	*Hypania invalida*	x	x		
Arthropoda					
Branchiopoda	*Cercopagis pengoi*		x	x	
	*Cornigerius maeoticus*		x		
	*Evadne anonyx*		x		
Malacostraca	*Chelicorophium (=Corophium) curvispinum*	x	x		
	*Chelicorophium robustum*	x			
	*Dikerogammarus haemobaphes*	x	x		
	*Dikerogammarus villosus*	x	x		
	*Echinogammarus ischnus*		x	x	x
	*Echinogammarus warpachowskyi*		x		
	*Hemimysis anomala*	x	x	x	
	*Jaera istri*	x			
	*Limnomysis benedeni*		x		
	*Obesogammarus crassus*	x	x		
	*Paramysis (=Mesomysis) intermedia*		x		
	*Paramysis (=Serrapalpisis) lacustris*		x		
	*Pontogammarus robustoides*		x		
	*Pseudocuma (=Stenocuma) graciloides*		x		
Maxillopoda	*Eurytemora affinis*			x	x
	*Nitocra hibernica*			x	
	*Nitocra incerta*			x	
	*Schizopera borutzkyi*			x	
Bryozoa					
Gymnolaemata	*Victorella pavida*	x	x		
Chordata					
Actinopterygii	*Acipenser gueldenstaedtii*	x	x		
	*Acipenser oxyrinchus*		x		
	*Acipenser ruthenus*	x	x		
	*Acipenser stellatus*		x		
	*Cyprinus carpio*		x	x	x
	*Huso huso*		x		
	*Neogobius fluviatilis*	x	x		
	*Neogobius kessleri*	x			
	*Neogobius melanostomus*	x	x	x	x
	*Proterorhinus marmoratus*		x	x	
Cnidaria					
Hydrozoa	*Cordylophora caspia*	x	x	x	
	*Maeotias marginata*		x		
	*Moerisia (=Ostroumovia) inkermanica*	x			
	*Pachycordyle navis*	x	x		
Mollusca					
Bivalvia	*Dreissena rostriformis bugensis*	x	x	x	x
	*Dreissena polymorpha*	x	x	x	x
Gastropoda	*Lithoglyphus naticoides*		x		
	*Theodoxus pallasi*		x		
	*Viviparus acerosus*	x			
Myxozoa					
Myxosporea	*Sphaeromyxa sevastopoli*			x	
Platyhelminthes					
Trematoda	*Ichthyocotylurus pileatus*			x	
	*Neascus brevicaudatus*			x	
Chromista					
Cercozoa	*Psammonobiotus communis*			x	
Gromiidea	*Psammonobiotus dziwnowi*			x	
	*Psammonobiotus linearis*			x	
Ciliophora					
Phyllopharyngea	*Acineta nitocrae*			x	

**Figure 3:**
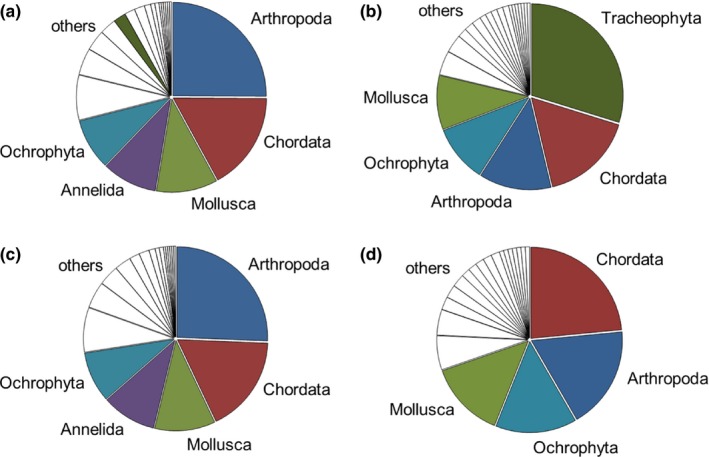
Phyla of aquatic nonindigenous species in the North and Baltic Seas including Tracheophyta (a) and excluding Tracheophyta (c) and in the Great Lakes–St. Lawrence River region including Tracheophyta (b) and excluding Tracheophyta (d). White fields in descending order show: Rhodophyta, Cnidaria, Myzozoa, Platyhelminthes, Bryozoa, Chlorophyta, Cercozoa, Ctenophora, Porifera, Acanthocephala, Ascomycota, Charophyta, Heterokontophyta, Nematoda, Proteobacteria (a and c), and Platyhelminthes, Annelida, Cercozoa, Chlorophyta, Virus, Cnidaria, Microsporidia, Myxozoa, Proteobacteria, Rhodophyta, Actinobacteria, Bryozoa, Charophyta, Ciliophora, Cyanobacteria, Euglenida, and Haptophyta (b and d)

Even though only 25 species established in both the North and Baltic Seas and Great Lakes–St. Lawrence River regions, the taxonomic composition of NIS was similar between the regions (Tables S1 and S2). The largest distinction was the phylum Tracheophyta; this phylum represented only 2% of species in the North and Baltic Seas region, but 30% in the Great Lakes–St. Lawrence River region. In the North and Baltic Seas region, the most abundant phyla were Arthropoda (25%, 69 species), Chordata (17%, 48 species), Mollusca (11%, 30 species), Annelida (10%, 27 species), and Ochrophyta (9%, 25 species), while those in the Great Lakes–St. Lawrence River region were Tracheophyta (30%, 56 species), Chordata (17%, 31 species), Arthropoda (13%, 24 species), Ochrophyta (10%, 19 species), and Mollusca (10%, 18 species; Figure 3). After excluding Tracheophyta from the datasets, the percentages of the most dominant phyla in the Great Lakes–St. Lawrence River region were 24, 18, 14, and 14% for Chordata, Arthropoda, Ochrophyta, and Mollusca, respectively (Figure 3).

Twenty‐four species that established in both the North and Baltic Seas region and the Great Lakes–St. Lawrence River region were Animalia, dominated by Actinopterygii (fishes); beside Animalia, there was one bacterium (*Aeromonas salmonicida*). Forty‐four percent of those species were from the Ponto‐Caspian region (eleven species; Tables 1, S1 and S2). More than two dozens of Arthropoda, Chordata, Cnidaria, and Mollusca that originated from the Ponto‐Caspian region and established in the North and Baltic Seas were not recorded in the Great Lakes–St. Lawrence River region. In contrast, Ponto‐Caspian Chromista established only in the Great Lakes–St. Lawrence River region (Tables 1, S1 and S2). Furthermore, taxonomic composition of NIS from Eurasia (after excluding Tracheophyta) was similar in the two regions, though only two species established in both regions (Tables S1 and S2).

### 3.2 Expected numbers of NIS and their comparison with observed numbers of NIS

**Figure 4:**
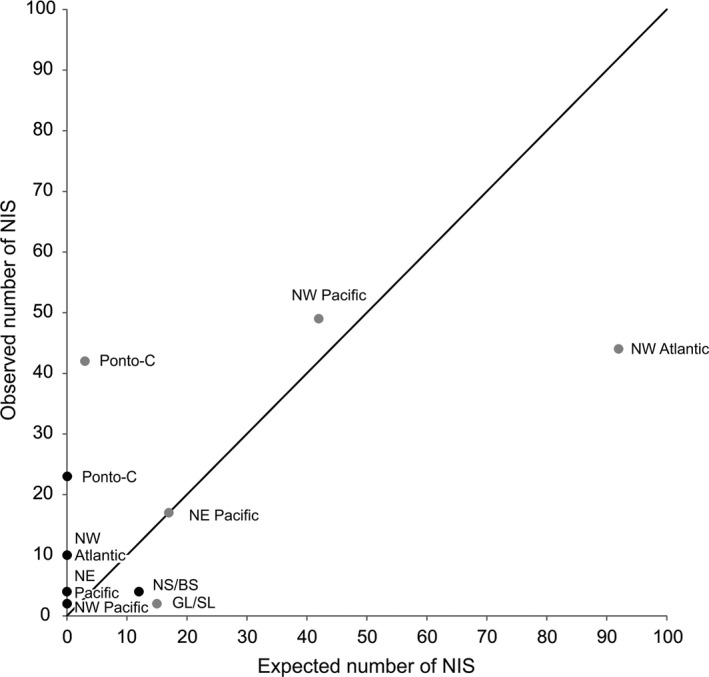
Scatter plot with number of expected nonindigenous species (NIS) in the North and Baltic Seas and the Great Lakes–St. Lawrence River region on *x*‐axis and number of observed NIS in these regions on *y*‐axis. Gray dots show the North and Baltic Seas region as the recipient region, and black dots show the Great Lakes–St. Lawrence River region as the recipient region. NW Pacific, NE Pacific, NW Atlantic, Ponto‐C, NS/BS, and GL/SL denote northwest Pacific, northeast Pacific, northwest Atlantic, Ponto‐Caspian region, North and Baltic Seas, and Great Lakes–St. Lawrence River region as donor regions of NIS, respectively. The line of unity is included

Estimated average native species richness for major donor regions ranged from 250 to 1,200 species (Table 2). Invasion risks between major donor regions and recipient regions varied greatly, with at least one or two orders of magnitude higher risks from donor regions to the North and Baltic Seas than to the Great Lakes–St. Lawrence River region. Expected numbers for both regions, the North and Baltic Seas and the Great Lakes–St. Lawrence River, were significantly different from observed numbers (chi‐square tests, *p* < .05). Estimated expected numbers of NIS from major donor regions were up to four orders of magnitude higher for the North and Baltic Seas than for the Great Lakes–St. Lawrence River region (Table 2). Observed numbers of NIS in the North and Baltic Seas from the northeast Pacific and northwest Pacific were similar to expected numbers from these regions (*p* > .5; Figure 4, Table 2). However, expected numbers of NIS from the northwest Atlantic and Great Lakes–St. Lawrence River region to the North and Baltic Seas were two and seven times higher, respectively, than observed numbers from these regions (*p* < .05; Figure 4, Table 2). The observed number of NIS from the Ponto‐Caspian region in the North and Baltic Seas was 14 times higher than expected (*p* < .05; Figure 4, Table 2). In the case of the Great Lakes–St.Lawrence River, observed numbers of NIS from the North and Baltic Seas were three times lower than expected numbers (*p* < .05; Figure 4, Table 2). The numbers of observed NIS in the Great Lakes–St.Lawrence River from all other donor regions were higher than expected ones, with Ponto‐Caspian species being more than 300 times higher (*p* < .05; Figure 4, Table 2).

**Table 2:** Estimated average species richness for major donor regions (per 880‐km resolution equal‐area grids), probabilities of invasion [*P*(Inv)] for species likely to be transported by ballast water from these regions and established in the North and Baltic Seas or the Great Lakes–St. Lawrence River region, estimated expected number of nonindigenous species (NIS) from major donor regions in the recipient regions, observed number of NIS from major donor regions in the recipient regions, and statistical comparisons of expected and observed numbers of NIS (i.e., chi‐square and *p*‐values) are shown. Significant *p*‐values are presented in bold

Donor region	Recipient region	Estimated average species richness	Invasion risk [*P*(Inv)]	Expected number of NIS	Observed number of NIS	Statistical comparison	
						Chi‐square	*p*‐Value
Northwest Atlantic	North and Baltic Seas	~570	.16172	92	44	25.04	**<.001**
Great Lakes–St. Lawrence River	North and Baltic Seas	~320	.04665	15	2	11.27	**<.001**
Northeast Pacific	North and Baltic Seas	~450	.03669	17	17	0	1
Northwest Pacific	North and Baltic Seas	~1,200	.03509	42	49	1.17	.279
Ponto‐Caspian region	North and Baltic Seas	**~**500	.00514	3	42	507	**<.001**
North and Baltic Seas	Great Lakes–St. Lawrence River	~250	.04849	12	4	5.33	**.021**
Ponto‐Caspian region	Great Lakes–St. Lawrence River	**~**500	.00014	0.07	23	7511	**<.001**
Northwest Atlantic	Great Lakes–St. Lawrence River	~570	.00006	0.03	10	3313	**<.001**
Northwest Pacific	Great Lakes–St. Lawrence River	~1,200	.00003	0.04	2	96.04	**<.001**
Northeast Pacific	Great Lakes–St. Lawrence River	~450	.00001	0.005	4	3192	**<.001**

## 4 Discussion

Several studies pointed out that species evolved in the Ponto‐Caspian region may be evolutionary predisposed to become NIS (Bij de Vaate et al., 2002; Leppäkoski et al., 2002, 2010; Reid & Orlova, 2002; Ricciardi & MacIsaac, 2000); however, the origin of NIS established in different regions have rarely been compared to confirm these statements. In this study, we explored origin of NIS established in the North and Baltic Seas and Great Lakes–St. Lawrence River regions—two areas intensively studied in concern to NIS, highly invaded by Ponto‐Caspian species, and with different salinity patterns (marine vs. freshwater). We compared established (observed) to expected numbers of NIS from donor regions and confirmed that there are many more Ponto‐Caspian species in both regions than expected based on the available pool of species from the Ponto‐Caspian region, frequency of shipping transits and an environmental match between regions. Additional vectors, in particular the canals that connect the North and Baltic Seas with the Black and Caspian Seas, might explain a higher number of Ponto‐Caspian taxa in northern Europe (this study; Bij de Vaate et al., 2002). Although, interestingly, the North and Baltic Seas are not dominant donor areas of NIS in the Ponto‐Caspian region (Shiganova, 2011). Contrarily, there is no such vector that would explain two orders of magnitude higher observed than expected numbers of Ponto‐Caspian taxa in the Great Lakes–St. Lawrence River. Previous studies suggested that the most probable pathway for Ponto‐Caspian species to the Great Lakes is a secondary introduction from northern Europe (Leppäkoski et al., 2002; Ricciardi & MacIsaac, 2000), though some species such as the quagga mussel *Dreissena rostriformis bugensis* likely came to the Great Lakes directly from the Black, Azov, or Caspian Sea (Spidle, Marsden, & May, 1994). As half of the Ponto‐Caspian species in the Great Lakes are not established in the North and Baltic Seas, the potential stepping stone dynamics via this region do not provide a parsimonious explanation. Northern European rivers however were not included in our study, and therefore, we cannot confidently disregard the stepping stone hypothesis. Species characteristics and their environmental tolerance, such as the production of dormant stages, r‐reproductive strategy (Briski, Ghabooli, Bailey, & MacIsaac, 2011; Briski, Ghabooli, & MacIsaac, 2012), or high phenotypic plasticity, may also explain colonization success of Ponto‐Caspian taxa under a lower propagule pressure scenario (i.e., low introduction effort).

Nevertheless, we show here that the Ponto‐Caspian region was one of the major donors for both the North and Baltic Seas and the Great Lakes–St. Lawrence River regions, and the main donor of NIS in both regions, only eleven of 56 species from that area established in both regions (i.e., 44 established in the North and Baltic Seas and 23 in the Great Lakes–St. Lawrence River). Bij de Vaate et al. (2002) described chronological spread for several Ponto‐Caspian species following the Danube and/or Dnieper rivers and migrating to the Rhine, Ems, Weser, Elbe, Oder, Vistula, and Neman rivers, with some of those species establishing in these rivers and their river mouths, but not spreading further to the North or Baltic Sea. In addition, the majority of Ponto‐Caspian NIS are established in the lower salinity areas of the Baltic, but not in the North Sea's higher salinity habitats (this study; Paavola, Olenin, & Leppäkoski, 2005). All the above mentioned may point to several directions: (1) even though the Ponto‐Caspian region is a donor region for both freshwater and marine habitats, all Ponto‐Caspian species are not able to equally thrive in both types of habitat; (2) Ponto‐Caspian species established in European rivers that are not in the North or Baltic Sea might spread to those areas in the future; (3) species established in Northern Europe, but not in the Great Lakes–St. Lawrence River region, might also reach the region in the future; (4) species established in the Great Lakes–St. Lawrence River but not in Northern Europe arrived to North America directly from the Ponto‐Caspian region, not as secondary introduction from Northern Europe; or (5) a combination of those above. To determine which of the above is correct or more significant, further studies on Ponto‐Caspian species, transport vectors, pathways, and propagule pressure regarding the Ponto‐Caspian area are needed.

Beside Ponto‐Caspian taxa, species from the northwest Atlantic, northwest and northeast Pacific were also established at higher numbers than expected in the Great Lakes–St. Lawrence River region. However, almost all of these species were intentionally introduced fishes (Ricciardi, 2006). In contrast, the number of established northwest Atlantic taxa in the North and Baltic Seas was lower than predicted. The number of the Great Lakes–St. Lawrence River taxa in the North and Baltic Seas, and vice versa, was also lower. The lower number of the North and Baltic Sea's taxa in the Great Lakes–St. Lawrence River region is quite intriguing as the shipping vector until the early 1980s was stronger than during 2007–2008 which were used for our probability estimations, and mainly unidirectional, delivering the Baltic water to the Great Lakes while transporting wheat to the USSR (Kelly, Lamberti, & MacIsaac, 2009). Underestimating the number of NIS might be a more common error due to unknown transport vectors that might operate between two regions, while overestimating is harder to explain because of a high propagule pressure between regions. An overestimated number of species from a certain region indicates that taxa from that region might be less suitable for colonization of new habitats. Therefore, taxa from the Great Lakes–St. Lawrence River, North and Baltic Seas, and northwest Atlantic seemed to be evolutionary less predisposed to be colonizers than Ponto‐Caspian taxa.

Previous studies stated that the transfer of species between different salinity habitats is asymmetrical, with a colonization of freshwater habitats by marine and brackish species becoming increasingly common in recent years, but not vice versa (Grigorovich, Pashkova, Gromova, & van Overdijk, 1998; Lee & Bell, 1999; Sylvester, Cataldo, Notaro, & Boltovskoy, 2013). Ponto‐Caspian species originating from brackish areas, with a salinity gradient from freshwater in the east to more saline in the west, accompanied by strong salinity fluctuations (Reid & Orlova, 2002) colonized both freshwater habitats of the Great Lakes and European major rivers, and brackish habitats of the St. Lawrence River and the North and Baltic Seas. However, the Ponto‐Caspian area is not an important donor of NIS to the Mediterranean Sea (CIESM, 2015). The Ponto‐Caspian region is geologically old and has several times undergone large‐scale environmental changes from fully marine environments, while it was a part of the Tethys Sea, to almost freshwater habitats as Sarmatian Sea (Reid & Orlova, 2002; Zenkevitch, 1963). Further, during the Pleistocene Epoch and the Ice Age, the majority of the Ponto‐Caspian area dried out, followed by freshwater flooding after ice melting at the end of the Ice Age. Later, a few more geological connections and disconnections of the whole or parts of the region with the Mediterranean Sea caused several additional changes in salinity, with Ponto‐Caspian low‐saline taxa surviving increases in salinity in surrounding rivers and spreading out to the basins once again when salinity dropped (Reid & Orlova, 2002; Zenkevitch, 1963). Hence, species evolved in this region may be freshwater taxa adapted to low‐saline habitats, accompanied later by Mediterranean taxa adapted to brackish environments. Therefore, colonization of the Great Lakes and European rivers by Ponto‐Caspian species should not be surprising. If endemic Ponto‐Caspian species are evolutionarily freshwater taxa, which are today highly euryhaline, then the statements about recent numerous colonizations of freshwater habitats by marine and brackish species are not surprising. However, further experimental and evolutionary studies are required to confirm this hypothesis.

The main difference in taxonomic composition of established NIS in the two regions was in the number of established plants. Tracheophyta was the most represented phylum of NIS established in the Great Lakes–St. Lawrence River (i.e., 30%, 56 species), most likely transported as seeds with solid ballast that was used prior to ballast water (e.g., sand, rocks and mud; Mills, Leach, Carlton, & Secor, 1993; Ricciardi, 2006). However, the phylum was negligible in the North and Baltic Sea region. Lambdon et al. (2008), taking into account the entire area of Europe, also stated that marine habitats are much less invaded by plants than inland waters. Beside the fact that the biodiversity of marine plants is much lower than that of freshwater plants (Les & Cleland, 1997), seeds of the latter are also highly resistant to harsh environments and drying conditions compared to those of the former (Cook, Gut, Rix, & Schneller, 1974; Larkum, Orth, & Duarte, 2007; Leck, 1989; Orth et al., 2000). Environmental tolerance, often dormancy of freshwater seeds, and dates of species discoveries support further the assumptions of solid ballast being the main vector for introduction of these taxa to aquatic habitats (Ricciardi, 2006). After replacement of solid ballast with ballast water at the beginning of the 20th Century, fewer introductions of plants were recorded in the Great Lakes (Ricciardi, 2006).

Taking into account numerous Ponto‐Caspian species established in the Great Lakes and Northern Europe, the areas which are greatly connected by shipping to Eastern Asia and coastal North America (Kaluza et al., 2010; Seebens et al., 2013), one would expect Ponto‐Caspian species spreading practically all around the world. However, as the Ponto‐Caspian region was not the main donor for the Mediterranean Sea (CIESM, 2015), nor did Ponto‐Caspian species establish in high salinities of the North Sea (this study; Paavola et al., 2005), we doubt that Ponto‐Caspian taxa may colonize highly saline marine habitats. We suspect that Ponto‐Caspian species would colonize big river mouths and estuaries, such as Chesapeake Bay, San Francisco Bay, Yangtze River, and Rio de la Plata. In addition to a salinity match among those regions and the Ponto‐Caspian region, very large shipping ports are also located in those areas. Comparative assessment of NIS in multiple regions around the world, including freshwater, brackish, and marine habitats, in connection with transport vectors (i.e., as a proxy for propagule pressure or introduction effort) and species characteristics would elucidate further if Ponto‐Caspian species are better colonizers than species evolved in other regions. However, this comparison would require a huge amount of work and sampling effort to establish reliable and complete lists of NIS, which are lacking for many areas around the world.

## Conflict of Interest

None declared.
