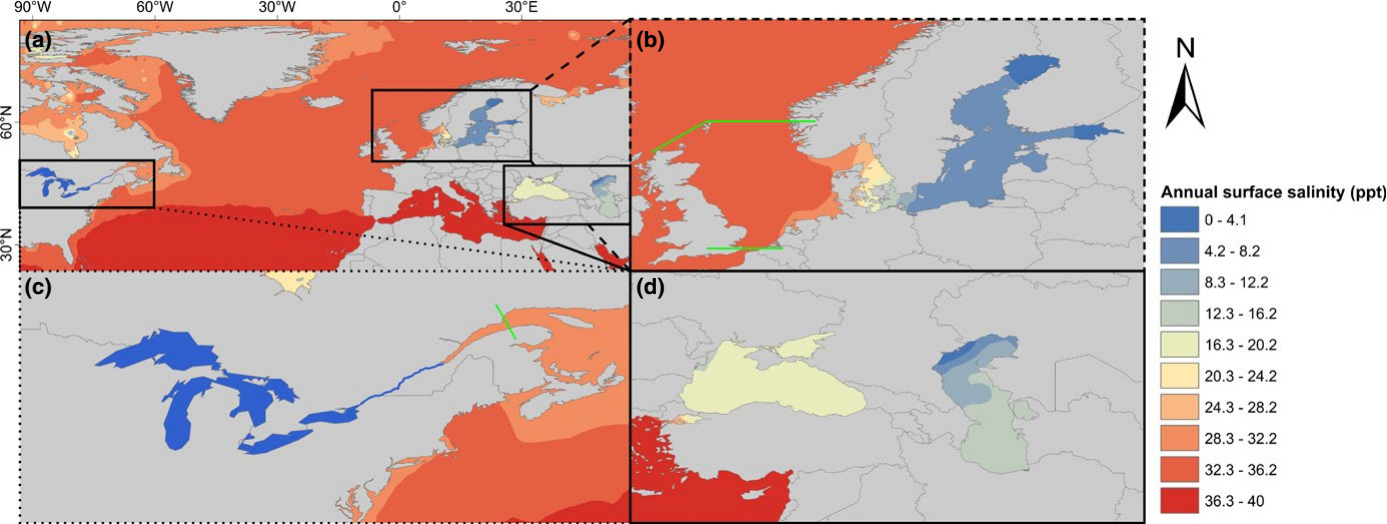# Corrigendum

**DOI:** 10.1002/ece3.3299

**Published:** 2017-08-29

Corrigendum to:

Casties I, Seebens H, Briski E (2016) Importance of geographic origin for invasion success: a case study of the North and Baltic Seas versus the Great Lakes‐St. Lawrence River region. Ecology and Evolution, 6(22), 8318–8329. https://doi.org/10.1002/ece3.2528

The authors of the paper Casties et al. (2016) want to note that Figure 1 appeared incorrectly. Please find the correct figure below.