# Supplementary material for: Importance of geographic origin for invasion success: A case study of the North and Baltic Seas versus the Great Lakes–St. Lawrence River region
Source: Ecol Evol. 2016 Oct 21;6(22):8318–29. doi: 10.1002/ece3.2528 (PMC5108280; doi:10.1002/ece3.2528)
Supplement: Supplementary file 1 [file ECE3-6-8318-s001.doc]

**Supporting Information**

Additional Supporting Information may be found in the online version of this article:

**APPENDIX S1** List of NIS established in the North and Baltic Seas, their geographic origin, and taxonomic assignment.

**APPENDIX S2** List of NIS established in the Great Lakes-St. Lawrence River region, their geographic origin, and taxonomic assignment.

**APPENDIX S1** List of NIS established in the North and Baltic Seas, their geographic origin, and taxonomic assignment. Asterisks mark the NIS that occur in both the North and Baltic Seas and the Great Lakes-St. Lawrence River regions. NS, BS, NW, NE, SW and SE denote the North Sea, Baltic Sea, north-west, north-east, south-west, and south-east, respectively. Eurasia represents inland freshwaters except Yangtze River, Indo-Pacific represents Indian Ocean and the archipelago of Indonesia, Malaysia, and Pilipinas, North America (N America) represents inland freshwaters except the Laurentian Great Lakes, St. Lawrence and Mississippi Rivers, while Australia, New Zealand, Africa and South America (S America) cover all inland freshwaters in these areas.

| **Taxon** | **Species** | **Invaded areas** | **Origin** |
| --- | --- | --- | --- |
| **Animalia** |  |  |  |
| **Acanthocephala** |  |  |  |
| **Eoacanthocephala** | *Paratenuisentis ambiguus* | BS | NE Pacific, NW Atlantic |
| **Annelida** |  |  |  |
| **Clitellata** | *Branchiura sowerbyi** | NS, BS | Eurasia |
|  | *Limnodrilus cervix* | BS | N America |
|  | *Paranais botniensis* | NS | unknown |
|  | *Paranais frici* | BS | Ponto-Caspian |
|  | *Potamothrix bedoti** | BS | Ponto-Caspian |
|  | *Potamothrix heuscheri* | BS | Ponto-Caspian |
|  | *Potamothrix vejdovskyi** | BS | Ponto-Caspian |
|  | *Tubificoides pseudogaster* | BS | North Sea |
| **Polychaeta** | *Alitta succinea* | BS | NE Atlantic |
|  | *Boccardia proboscidea* | NS | NE Pacific |
|  | *Boccardiella ligerica* | NS, BS | unknown |
|  | *Clymenella torquata* | NS | NW Atlantic |
|  | *Ficopomatus enigmaticus* | NS, BS | Indo-Pacific |
|  | *Hydroides dianthus* | NS | NW Atlantic |
|  | *Hydroides elegans* | NS | unknown |
|  | *Hydroides ezoensis* | NS | NW Pacific |
|  | *Hypania invalida* | NS, BS | Ponto-Caspian |
|  | *Laonome calida* | NS | unknown |
|  | *Marenzelleria arctia* | BS | Arctic |
|  | *Marenzelleria neglecta* | NS, BS | NW Atlantic |
|  | *Marenzelleria viridis* | NS, BS | NW Atlantic |
|  | *Marenzelleria wireni* | NS | NW Atlantic |
|  | *Marphysa sanguinea* | NS | unknown |
|  | *Neodexiospira (=Janua) brasiliensis* | NS | SW Atlantic |
|  | *Pileolaria berkeleyana* | NS | NW Pacific |
|  | *Scolelepis bonnieri* | NS | unknown |
|  | *Tharyx killariensis* | NS | unknown |
| **Arthropoda** |  |  |  |
| **Branchiopoda** | *Cercopagis pengoi** | BS | Ponto-Caspian |
|  | *Cornigerius maeoticus* | BS | Ponto-Caspian |
|  | *Evadne anonyx* | BS | Ponto-Caspian |
|  | *Penilia avirostris* | NS, BS | unknown |
| **Insecta** | *Telmatogeton japonicus* | NS, BS | NW Pacific |
| **Malacostraca** | *Atyaephyra desmarestii* | NS | Eurasia |
|  | *Brachynotus sexdentatus* | NS | NE Atlantic, SE Atlantic |
|  | *Callinectes sapidus* | NS, BS | NW Atlantic |
|  | *Caprella mutica* | NS | NW Pacific |
|  | *Chelicorophium (=Corophium) curvispinum* | NS, BS | Ponto-Caspian |
|  | *Chelicorophium robustum* | NS | Ponto-Caspian |
|  | *Dikerogammarus haemobaphes* | NS, BS | Ponto-Caspian |
|  | *Dikerogammarus villosus* | NS, BS | Ponto-Caspian |
|  | *Echinogammarus ischnus** | BS | Ponto-Caspian |
|  | *Echinogammarus warpachowskyi* | BS | Ponto-Caspian |
|  | *Eriocheir sinensis* | NS, BS | NW Pacific |
|  | *Gammarus tigrinus** | NS, BS | NW Atlantic |
|  | *Gmelinoides fasciatus* | BS | Eurasia |
|  | *Goneplax rhomboides* | NS | NE Atlantic |
|  | *Hemigrapsus penicillatus* | NS | NW Pacific |
|  | *Hemigrapsus sanguineus* | NS | NW Pacific |
|  | *Hemigrapsus takanoi* | NS | NW Pacific |
|  | *Hemimysis anomala** | NS, BS | Ponto-Caspian |
|  | *Homarus americanus* | NS, BS | NW Atlantic |
|  | *Ianiropsis tridens* | NS | unknown |
|  | *Incisocalliope aestuarius* | NS | NW Atlantic |
|  | *Jaera istri* | NS | Ponto-Caspian |
|  | *Jassa marmorata* | NS | NW Atlantic, Mediterranean |
|  | *Limnomysis benedeni* | BS | Ponto-Caspian |
|  | *Melita nitida* | NS, BS | NW Atlantic |
|  | *Monocorophium sextonae* | NS | unknown |
|  | *Monocorophium uenoi* | NS | unknown |
|  | *Obesogammarus crassus* | NS, BS | Ponto-Caspian |
|  | *Orchestia cavimana* | BS | unknown |
|  | *Orconectes limosus** | BS | N America |
|  | *Orconectes virilis* | NS | N America |
|  | *Palaemon elegans* | BS | NW Atlantic |
|  | *Palaemon macrodactylus* | NS | NW Pacific |
|  | *Paramysis (=Mesomysis) intermedia* | BS | Ponto-Caspian |
|  | *Paramysis (=Serrapalpisis) lacustris* | BS | Ponto-Caspian |
|  | *Platorchestia platensis* | NS, BS | SW Atlantic |
|  | *Pontogammarus robustoides* | BS | Ponto-Caspian |
|  | *Proasellus coxalis* | NS | Mediterranean |
|  | *Procambarus fallax* f. *virginalis* | NS | unknown |
|  | *Pseudocuma (=Stenocuma) graciloides* | BS | Ponto-Caspian |
|  | *Rhithropanopeus harrisii* | NS, BS | NW Atlantic |
|  | *Sinelobus stanfordi* | NS | unknown |
|  | *Synidotea laticauda* | NS | NE Pacific |
| **Maxillopoda** | *Acartia tonsa* | NS, BS | NW Atlantic, Indo-Pacific |
|  | *Ameira divagans divagans* | NS, BS | NW Atlantic |
|  | *Amphibalanus (=Balanus) improvisus* | NS, BS | NW Atlantic |
|  | *Amphibalanus eburneus* | NS | NW Atlantic |
|  | *Amphibalanus reticulatus* | NS | Indo-Pacific |
|  | *Amphibalanus variegatus* | NS | Indo-Pacific |
|  | *Austrominius modestus* | NS | SW Pacific |
|  | *Balanus amphitrite* | NS | Indo-Pacific |
|  | *Balanus trigonus* | NS | unknown |
|  | *Conchoderma auritum* | NS | unknown |
|  | *Eurytemora americana* | NS | NW Atlantic |
|  | *Megabalanus coccopoma* | NS | SE Pacific |
|  | *Megabalanus tintinnabulum* | NS | unknown |
|  | *Myicola ostreae* | NS | NW Pacific |
|  | *Mytilicola intestinalis* | NS | Mediterranean |
|  | *Mytilicola orientalis (=ostreae)* | NS | NW Pacific |
|  | *Skistodiaptomus pallidus** | NS | N America |
|  | *Solidobalanus fallax* | BS | SE Atlantic |
| **Merostomata** | *Limulus polyphemus* | NS, BS | NW Atlantic |
| **Ostracoda** | *Eusarsiella zostericola* | NS | NW Atlantic |
| **Pycnogonida** | *Ammothea hilgendorfi* | NS | NW Pacific |
| **Bryozoa** |  |  |  |
| **Gymnolaemata** | *Bugula neritina* | NS | NW Atlantic |
|  | *Bugula stolonifera* | NS | NW Atlantic |
|  | *Smittoidea prolifica* | NS | NE Pacific, NW Pacific |
|  | *Tricellaria inopinata* | NS | unknown |
|  | *Victorella pavida* | NS, BS | Ponto- Caspian |
| **Chordata** |  |  |  |
| **Actinopterygii** | *Acipenser baerii* | NS, BS | Eurasia |
|  | *Acipenser gueldenstaedtii* | NS, BS | Ponto-Caspian |
|  | *Acipenser oxyrinchus* | BS | Ponto-Caspian |
|  | *Acipenser ruthenus* | NS, BS | Ponto-Caspian |
|  | *Acipenser stellatus* | BS | Ponto-Caspian |
|  | *Acipenser transmontanus* | NS | NE Pacific |
|  | *Carassius gibelio* | BS | unknown |
|  | *Catostomus catostomus* | BS | N America |
|  | *Coregonus autumnalis* | BS | Arctic |
|  | *Coregonus muksun* | BS | Eurasia |
|  | *Coregonus nasus* | BS | Eurasia |
|  | *Coregonus peled* | BS | Eurasia |
|  | *Ctenopharyngodon idella* | BS | Eurasia |
|  | *Cyprinus carpio** | BS | Ponto-Caspian |
|  | *Huso huso* | BS | Ponto-Caspian |
|  | *Hypophthalmichthys molitrix* | NS, BS | Eurasia |
|  | *Hypophthalmichthys nobilis* | BS | Eurasia |
|  | *Lepomis gibbosus* | NS, BS | N America |
|  | *Micropogonias undulatus* | NS | NW Atlantic |
|  | *Micropterus dolomieu* | NS, BS | Great Lakes |
|  | *Micropterus salmoides* | BS | Great Lakes |
|  | *Morone saxatilis* | NS | N America |
|  | *Neogobius fluviatilis* | NS, BS | Ponto-Caspian |
|  | *Neogobius kessleri* | NS | Ponto-Caspian |
|  | *Neogobius melanostomus** | NS, BS | Ponto-Caspian |
|  | *Oncorhynchus clarkii** | NS | NE Pacific |
|  | *Oncorhynchus gorbuscha** | BS | NE Pacific, NW Pacific |
|  | *Oncorhynchus keta* | BS | NE Pacific, NW Pacific |
|  | *Oncorhynchus kisutch** | NS, BS | NE Pacific, NW Pacific |
|  | *Oncorhynchus mykiss** | NS, BS | NE Pacific |
|  | *Oncorhynchus nerka** | BS | NE Pacific, NW Pacific |
|  | *Oncorhynchus tshawytscha** | BS | NE Pacific, NW Pacific |
|  | *Oreochromis niloticus niloticus* | NS | Africa |
|  | *Perccottus glenii* | BS | NW Pacific |
|  | *Poecilia (=Lebistes) reticulata* | NS | NW Atlantic |
|  | *Proterorhinus marmoratus** | BS | Ponto-Caspian |
|  | *Salvelinus fontinalis* | NS, BS | N America |
|  | *Salvelinus namaycush* | NS, BS | N America |
|  | *Sander lucioperca* | NS | Eurasia |
|  | *Sebastes schlegelii* | NS | NW Pacific, SW Pacific |
|  | *Tetraodon fluviatilis* | NS | Eurasia |
|  | *Trinectes maculatus* | NS | NW Atlantic |
| **Ascidiacea** | *Aplidium glabrum* | NS | unknown |
|  | *Botrylloides violaceus* | NS | NW Pacific |
|  | *Didemnum vexillum* | NS | NW Pacific |
|  | *Perophora japonica* | NS | unknown |
|  | *Styela clava* | NS, BS | NW Pacific |
| **Aves** | *Branta canadensis* | BS | N America |
| **Cnidaria** |  |  |  |
| **Anthozoa** | *Diadumene cincta* | NS | NE Pacific, NW Pacific |
|  | *Diadumene lineata* | NS | NW Pacific |
|  | *Nematostella vectensis* | NS | NW Atlantic |
| **Hydrozoa** | *Bougainvillia macloviana* | NS | unknown |
|  | *Bougainvillia rugosa* | BS | NE Pacific, NW Atlantic |
|  | *Cordylophora caspia** | NS, BS | Ponto-Caspian |
|  | *Craspedacusta sowerbii** | NS | Yangtze river |
|  | *Garveia franciscana* | NS | NW Atlantic |
|  | *Gonionemus vertens* | NS, BS | NW Pacific |
|  | *Maeotias marginata* | BS | Ponto-Caspian |
|  | *Moerisia (=Ostroumovia) inkermanica* | NS | Ponto-Caspian |
|  | *Nemopsis bachei* | NS | NW Atlantic |
|  | *Pachycordyle navis* | NS, BS | Ponto-Caspian |
| **Ctenophora** |  |  |  |
| **Nuda** | *Beroe ovata* | BS | NW Atlantic |
| **Tentaculata** | *Mnemiopsis leidyi* | NS, BS | NW Atlantic, SW Atlantic |
| **Mollusca** |  |  |  |
| **Bivalvia** | *Corbicula fluminea** | NS | Eurasia |
|  | *Crassostrea gigas* | NS, BS | NW Pacific |
|  | *Crassostrea virginica* | NS, BS | NW Atlantic |
|  | *Dreissena rostriformis bugensis** | NS, BS | Ponto-Caspian |
|  | *Dreissena polymorpha** | NS, BS | Ponto-Caspian |
|  | *Ensis directus (=americanus)* | NS, BS | NW Atlantic |
|  | *Mercenaria mercenaria* | NS | NW Atlantic |
|  | *Mya arenaria* | NS, BS | NW Atlantic |
|  | *Mytilopsis leucophaeata* | NS, BS | NW Atlantic |
|  | *Petricolaria pholadiformis* | NS, BS | NW Atlantic |
|  | *Psiloteredo megotara* | NS | unknown |
|  | *Rangia cuneata* | NS, BS | NW Atlantic |
|  | *Ruditapes philippinarum* | NS | NW Pacific |
|  | *Spisula solidissima* | NS | NW Atlantic |
|  | *Teredo navalis* | NS, BS | Indo-Pacific |
| **Gastropoda** | *Bellamya chinensis* | NS | Eurasia |
|  | *Corambe obscura (=batava)* | NS | NW Atlantic |
|  | *Crepidula fornicata* | NS, BS | NW Atlantic |
|  | *Ferrissia wautieri* | NS | unknown |
|  | *Hexaplex (Trunculariopsis) trunculus* | NS | unknown |
|  | *Lithoglyphus naticoides* | BS | Ponto-Caspian |
|  | *Ocenebra erinaceus* | NS | unknown |
|  | *Ocenebra inornata* | NS | unknown |
|  | *Physella acuta* | NS | Eurasia |
|  | *Potamopyrgus antipodarum** | NS, BS | New Zealand |
|  | *Rapana venosa* | NS | NW Pacific |
|  | *Theodoxus pallasi* | BS | Ponto-Caspian |
|  | *Urosalpinx cinerea* | NS | NW Atlantic |
|  | *Viviparus acerosus* | NS | Ponto-Caspian |
|  | *Viviparus viviparus* | NS | unknown |
| **Nematoda** |  |  |  |
| **Chromadorea** | *Anguillicola crassus* | NS, BS | Eurasia |
| **Platyhelminthes** |  |  |  |
| **Cestoda** | *Dugesia tigrina* | NS | N America |
| **Monogenea** | *Gyrodactylus salaris* | NS | Baltic Sea |
|  | *Onchocleidus dispar* | NS | NW Atlantic |
|  | *Pseudodactylogyrus anguillae* | NS, BS | NW Pacific |
|  | *Pseudodactylogyrus bini* | NS, BS | NW Pacific |
| **Trematoda** | *Pseudobacciger harengulae* | NS | NW Pacific |
| **Turbellaria** | *Euplana gracilis* | NS | unknown |
|  | *Imogine necopinata* | NS | unknown |
| **Porifera** |  |  |  |
| **Demospongiae** | *Celtodoryx ciocalyptoides* | NS | unknown |
|  | *Chalinula loosanoffi* | NS | NE Atlantic |
| **Bacteria** |  |  |  |
| **Proteobacteria** | *Aeromonas salmonicida** | NS | unknown |
| **Chromista** |  |  |  |
| **Cercozoa** |  |  |  |
| **Ascetosporea** | *Bonamia ostreae* | NS | unknown |
|  | *Haplosporidium armoricanum* | NS | NE Atlantic, SE Atlantic |
|  | *Marteilia refringens* | NS | NE Atlantic, SE Atlantic |
| **Myzozoa** |  |  |  |
| **Dinophyceae** | *Alexandrium leeii* | NS | NE Pacific, NW Pacific |
|  | *Dicroerisma psilonereiella* | NS | unknown |
|  | *Gymnodinium aureolum* | NS | NE Pacific, NW Pacific |
|  | *Gymnodinium catenatum* | NS | unknown |
|  | *Karenia mikimotoi* | NS, BS | NW Pacific |
|  | *Karlodinium veneficum* | NS | unknown |
|  | *Oxytoxum criophilum* | NS | unknown |
|  | *Pfiesteria piscicida* | NS | NE Pacific, NW Atlantic |
|  | *Pfiesteria shumwayae* | NS | unknown |
|  | *Thecadinium yashimaense* | NS | NE Pacific, NW Pacific |
| **Ochrophyta** |  |  |  |
| **Bacillariophyceae** | *Coscinodiscus wailesii* | NS, BS | Indo-Pacific |
|  | *Lennoxia faveolata* | BS | unknown |
|  | *Odontella sinensis* | NS, BS | Indo-Pacific |
|  | *Pleurosigma simonsenii* | NS | Indian Ocean |
|  | *Stephanopyxis palmeriana* | NS | unknown |
|  | *Thalassiosira hendeyi* | NS | SE Atlantic, SW Atlantic |
|  | *Thalassiosira punctigera* | NS, BS | NE Pacific, NW Pacific |
|  | *Thalassiosira tealata* | NS | NE Pacific, NW Pacific |
| **Phaeophyceae** | *Asperococcus scaber* | NS | unknown |
|  | *Botrytella* sp. | NS | unknown |
|  | *Colpomenia peregrina* | NS, BS | NE Pacific |
|  | *Corynophlaea umbellata* | NS | NE Pacific, NW Pacific |
|  | *Corynophlaea verruculiformis* | NS | NW Pacific |
|  | *Elachista* sp. | NS | unknown |
|  | *Fucus evanescens* | NS, BS | Arctic |
|  | *Leathesia verruculiformis* | NS | unknown |
|  | *Myriactula* sp. | NS | unknown |
|  | *Saccharina japonica* | NS | unknown |
|  | *Sargassum muticum* | NS, BS | NW Pacific |
|  | *Scytosiphon dotyi* | NS | unknown |
|  | *Undaria pinnatifida* | NS | NW Pacific |
| **Raphidophyceae** | *Chattonella marina* | NS | unknown |
|  | *Chattonella marina* var. *antiqua* | NS | NE Pacific, NW Pacific |
|  | *Chattonella verruculosa* | NS | NW Pacific |
|  | *Fibrocapsa japonica* | NS | NE Pacific, NW Pacific |
|  | *Heterosigma akashiwo (carterae)* | NS | NW Pacific |
| **Fungi** |  |  |  |
| **Ascomycota** |  |  |  |
| **Sordariomycetes** | *Claviceps purpurea* | NS | unknown |
| **Plantae** |  |  |  |
| **Charophyta** |  |  |  |
| **Charophyceae** | *Chara connivens* | BS | Eurasia |
| **Chlorophyta** |  |  |  |
| **Bryopsidophyceae** | *Codium fragile atlanticum* | NS | Indo Pacific |
|  | *Codium fragile fragile (tomentosoides)* | NS, BS | NW Pacific |
|  | *Codium fragile ssp. scandinavicum* | NS | Indo-Pacific |
| **Ulvophyceae** | *Protomonostroma undulatum* | BS | Indo-Pacific |
|  | *Ulva pertusa* | NS | NW Pacific |
| **Rhodophyta** |  |  |  |
| **Florideophyceae** | *Acrochaetium catenulatum* | NS | NE Pacific, NW Pacific |
|  | *Acrochaetium savianum* | NS | NW Atlantic |
|  | *Agardhiella subulata* | NS | unknown |
|  | *Aglaothamnion halliae* | NS | NW Atlantic |
|  | *Anotrichium furcellatum* | NS | Mediterranean |
|  | *Antithamnion nipponicum* | NS | NW Pacific |
|  | *Antithamnionella spirographidis* | NS | NW Pacific |
|  | *Antithamnionella ternifolia* | NS | SW Pacific |
|  | *Asparagopsis armata* | NS | SW Pacific |
|  | *Bonnemaisonia hamifera* | NS, BS | NW Pacific |
|  | *Ceramium cimbricum* | NS | unknown |
|  | *Colaconema dasyae* | NS | unknown |
|  | *Dasya baillouviana* | NS, BS | NE Atlantic, Mediterranean |
|  | *Dasysiphonia* sp. | NS | NW Pacific |
|  | *Devaleraea ramentacea* | NS | unknown |
|  | *Gracilaria vermiculophylla* | NS, BS | NW Pacific |
|  | *Grateloupia doryphora* | NS | NE Pacific |
|  | *Grateloupia luxurians* | NS | Indo-Pacific |
|  | *Heterosiphonia japonica* | NS, BS | NW Pacific |
|  | *Lomentaria hakodatensis* | NS | NE Pacific, NW Pacific |
|  | *Neosiphonia harveyi* | NS, BS | NW Pacific |
|  | *Polysiphonia senticulosa* | NS | NW Pacific |
| **Tracheophyta** |  |  |  |
| **Liliopsida** | *Elodea canadensis* | BS | N America |
|  | *Elodea nuttallii* | NS | N America |
|  | *Spartina anglica* | NS, BS | NE Atlantic |
|  | *Spartina townsendii* | NS, BS | NE Atlantic |
| **Magnoliopsida** | *Cotula coronopifolia* | NS | SE Atlantic |
|  | *Ludwigia grandiflora* (Michx.) | NS | unknown |

**APPENDIX S2** List of NIS established in the Great Lakes-St. Lawrence River region, their geographic origin, and taxonomic assignment. Asterisks mark the NIS that occur in both the North and Baltic Seas and the Great Lakes-St. Lawrence River regions. GL, SL, NW, NE, SW and SE denote the Great Lakes, St. Lawrence River, north-west, north-east, south-west, and south-east, respectively. Eurasia represents inland freshwaters except Yangtze River, Indo-Pacific represents Indian Ocean and the archipelago of Indonesia, Malaysia, and Pilipinas, North America (N America) represents inland freshwaters except the Laurentian Great Lakes, St. Lawrence and Mississippi Rivers, while Australia, New Zealand, Africa and South America (S America) cover all inland freshwaters in these areas.

| **Taxon** | **Species** | **Invaded areas** | **Origin** |
| --- | --- | --- | --- |
| **Animalia** |  |  |  |
| **Annelida** |  |  |  |
| **Clitellata** | *Branchiura sowerbyi** | GL | Eurasia |
|  | *Gianius aquaedulcis* | GL | Eurasia |
|  | *Potamothrix bedoti** | GL | Ponto-Caspian |
|  | *Potamothrix moldaviensis* | GL | Ponto-Caspian |
|  | *Potamothrix vejdovskyi** | GL | Ponto-Caspian |
|  | *Ripistes parasita* | GL, SL | Eurasia |
| **Arthropoda** |  |  |  |
| **Branchiopoda** | *Bosmina maritima* | GL | Baltic Sea |
|  | *Bythotrephes longimanus* | GL | Eurasia |
|  | *Cercopagis pengoi** | GL | Ponto-Caspian |
|  | *Daphnia galeata galeata* | GL | Arctic |
|  | *Daphnia lumholtzi* | GL | Australia |
|  | *Eubosmina coregoni* | GL, SL | Eurasia |
| **Insecta** | *Acentropus niveus* | GL | Eurasia |
|  | *Tanysphyrus lemnae* | GL | Eurasia |
| **Malacostraca** | *Echinogammarus ischnus** | GL, SL | Ponto-Caspian |
|  | *Gammarus tigrinus** | GL | NW Atlantic |
|  | *Hemimysis anomala** | GL | Ponto-Caspian |
|  | *Orconectes limosus** | SL | N America |
|  | *Orconectes rusticus* | GL | N America |
| **Maxillipoda** | *Argulus japonicus* | GL | Eurasia |
|  | *Cyclops strenuus* | GL | unknown |
|  | *Eurytemora affinis* | GL, SL | Ponto-Caspian |
|  | *Heteropsyllus nr. nunni* | GL | unknown |
|  | *Megacyclops viridis* | GL | Eurasia |
|  | *Neoergasilus japonicus* | GL | Eurasia |
|  | *Nitocra hibernica* | GL | Ponto-Caspian |
|  | *Nitocra incerta* | GL | Ponto-Caspian |
|  | *Salmincola lotae* | GL | Eurasia |
|  | *Schizopera borutzkyi* | GL | Ponto-Caspian |
|  | *Skistodiaptomus pallidus** | GL | N America |
| **Bryozoa** |  |  |  |
| **Phylactolaemata** | *Lophopodella carteri* | GL, SL | Eurasia, Africa |
| **Chordata** |  |  |  |
| **Actinopterygii** | *Alosa aestivalis* | GL | NW Atlantic |
|  | *Alosa pseudoharengus* | GL | NW Atlantic |
|  | *Apeltes quadracus* | GL | NE Atlantic, NW Atlantic |
|  | *Carassius auratus* | GL, SL | Eurasia |
|  | *Cyprinus carpio** | GL, SL | Ponto-Caspian |
|  | *Enneacanthus gloriosus* | GL | N America |
|  | *Esox niger* | GL | NW Atlantic |
|  | *Gambusia affinis* | GL | N America |
|  | *Gymnocephalus cernuus* | GL | Eurasia |
|  | *Ictiobus cyprinellus* | GL | N America |
|  | *Lepisosteus platostomus* | GL | Mississippi River |
|  | *Lepomis humilis* | GL | N America |
|  | *Lepomis microlophus* | GL | N America |
|  | *Misgurnus anguillicaudatus* | GL | Eurasia |
|  | *Morone americana* | GL | N America |
|  | *Neogobius melanostomus** | GL, SL | Ponto-Caspian |
|  | *Notropis buchanani* | GL | Mississippi River |
|  | *Noturus insignis* | GL, SL | N America |
|  | *Oncorhynchus clarkii** | SL | NE Pacific |
|  | *Oncorhynchus gorbuscha** | GL | NE Pacific, NW Pacific |
|  | *Oncorhynchus kisutch** | GL, SL | NE Pacific, NW Pacific |
|  | *Oncorhynchus mykiss** | GL, SL | NE Pacific |
|  | *Oncorhynchus nerka** | GL | NE Pacific, NW Pacific |
|  | *Oncorhynchus tshawytscha** | GL, SL | NE Pacific, NW Pacific |
|  | *Osmerus mordax* | GL | N America |
|  | *Phenacobius mirabilis* | GL | Mississippi River |
|  | *Proterorhinus marmoratus** | GL | Ponto-Caspian |
|  | *Salmo trutta* | GL, SL | Eurasia |
|  | *Scardinius erythrophthalmus* | GL, SL | Eurasia |
|  | *Tinca tinca* | SL | Eurasia |
| **Petromyzonti** | *Petromyzon marinus* | GL | N America |
| **Cnidaria** |  |  |  |
| **Hydrozoa** | *Cordylophora caspia** | GL | Ponto-Caspian |
|  | *Craspedacusta sowerbii** | GL | Yangtze River |
| **Mollusca** |  |  |  |
| **Bivalvia** | *Corbicula fluminea** | GL | Eurasia |
|  | *Dreissena rostriformis bugensis** | GL, SL | Ponto-Caspian |
|  | *Dreissena polymorpha** | GL, SL | Ponto-Caspian |
|  | *Lasmigona subviridis* | GL | N America |
|  | *Pisidium amnicum* | GL, SL | Eurasia |
|  | *Pisidium henslowanum* | GL, SL | Eurasia |
|  | *Pisidium moitessierianum* | GL | Eurasia |
|  | *Pisidium supinum* | GL | Eurasia |
|  | *Sphaerium corneum* | GL, SL | Eurasia |
| **Gastropoda** | *Bithynia tentaculata* | GL, SL | Eurasia |
|  | *Cipangopaludina japonica* | GL | Eurasia |
|  | *Cipangopaludina malleata* | GL, SL | Eurasia |
|  | *Elimia virginica* | GL | N America |
|  | *Gillia altilis* | GL | N America |
|  | *Potamopyrgus antipodarum** | GL | New Zealand |
|  | *Radix auricularia* | GL, SL | Eurasia |
|  | *Valvata piscinalis* | GL, SL | Eurasia |
|  | *Viviparus georgianus* | GL, SL | N America |
| **Myxozoa** |  |  |  |
| **Myxosporea** | *Myxobolus cerebralis* | GL | unknown |
|  | *Sphaeromyxa sevastopoli* | GL | Ponto-Caspian |
| **Platyhelminthes** |  |  |  |
| **Cestoda** | *Bothriocephalus acheilognathi* | GL | Eurasia |
|  | *Dugesia polychroa* | GL, SL | Eurasia |
|  | *Scolex pleuronectis* | GL | unknown |
| **Monogenea** | *Dactylogyrus amphibothrium* | GL | Eurasia |
|  | *Dactylogyrus hemiamphibothrium* | GL | Eurasia |
| **Trematoda** | *Ichthyocotylurus pileatus* | GL | Ponto-Caspian |
|  | *Neascus brevicaudatus* | GL | Ponto-Caspian |
|  | *Timoniella* sp. | GL | unknown |
| **Bacteria** |  |  |  |
| **Actinobacteria** | *Renibacterium salmoninarum* | GL | unknown |
| **Cyanobacteria** |  |  |  |
| **Cyanophyceae** | *Cylindrospermopsis raciborskii* | GL | unknown |
| **Proteobacteria** |  |  |  |
| **Gammaproteobacteria** | *Aeromonas salmonicida** | GL, SL | unknown |
|  | *Piscirickettsia* cf. *salmonis* | GL | unknown |
| **Chromista** |  |  |  |
| **Cercozoa** |  |  |  |
| **Gromiidea** | *Psammonobiotus communis* | GL | Ponto-Caspian |
|  | *Psammonobiotus dziwnowi* | GL | Ponto-Caspian |
|  | *Psammonobiotus linearis* | GL | Ponto-Caspian |
| **Ciliophora** |  |  |  |
| **Phyllopharyngea** | *Acineta nitocrae* | GL | Ponto-Caspian |
| **Haptophyta** |  |  |  |
| **Prymnesiophyceae** | *Hymenomonas roseola* | GL | Eurasia |
| **Ochrophyta** |  |  |  |
| **Bacillariophyceae** | *Actinocyclus normanii* fo*. subsalsa* | GL | Baltic Sea |
|  | *Biddulphia laevis* | GL | unknown |
|  | *Chaetoceros hohnii* | GL | unknown |
|  | *Cyclotella atomus* | GL | unknown |
|  | *Cyclotella cryptica* | GL | unknown |
|  | *Cyclotella pseudostelligera* | GL, SL | unknown |
|  | *Cyclotella woltereki* | GL | unknown |
|  | *Diatoma ehrenbergii* | GL, SL | unknown |
|  | *Skeletonema potamos* | GL, SL | unknown |
|  | *Skeletonema subsalsum* | GL, SL | Eurasia |
|  | *Stephanodiscus binderanus* | GL, SL | Baltic Sea |
|  | *Stephanodiscus subtilis* | GL | Eurasia |
|  | *Thalassiosira baltica* | GL | unknown |
|  | *Thalassiosira guillardii* | GL, SL | unknown |
|  | *Thalassiosira lacustris* | GL | unknown |
|  | *Thalassiosira pseudonana* | GL, SL | unknown |
|  | *Thalassiosira weissflogii* | GL, SL | unknown |
| **Phaeophyceae** | *Sphacelaria fluviatilis* | GL | Eurasia |
|  | *Sphacelaria lacustris* | GL | unknown |
| **Fungi** |  |  |  |
| **Microsporidia** |  |  |  |
| **Microsporea** | *Glugea hertwigi* | GL, SL | Baltic Sea |
|  | *Heterosporis* sp. | GL | unknown |
| **Plantae** |  |  |  |
| **Charophyta** |  |  |  |
| **Charophyceae** | *Nitellopsis obtusa* | GL, SL | Eurasia |
| **Chlorophyta** |  |  |  |
| **Ulvophyceae** | *Enteromorpha flexuosa* | GL | unknown |
|  | *Enteromorpha intestinalis* | GL, SL | NW Atlantic |
|  | *Enteromorpha prolifera* | GL, SL | unknown |
| **Rhodophyta** |  |  |  |
| **Bangiophyceae** | *Bangia atropurpurea* | GL | unknown |
| **Rhodellophyceae** | *Chroodactylon ramosum* | GL, SL | unknown |
| **Tracheophyta** |  |  |  |
| **Liliopsida** | *Agrostis gigantea* | GL, SL | Eurasia |
|  | *Alopecurus geniculatus* | GL, SL | Eurasia |
|  | *Butomus umbellatus* | GL, SL | Eurasia |
|  | *Carex acutiformis* | GL | Eurasia |
|  | *Carex disticha* | GL, SL | Eurasia |
|  | *Echinochloa crusgalli* | GL, SL | Eurasia |
|  | *Glyceria maxima* | GL | Eurasia |
|  | *Hydrocharis morsus-ranae* | GL, SL | Eurasia |
|  | *Iris pseudacorus* | GL, SL | Eurasia |
|  | *Juncus compressus* | GL, SL | Eurasia |
|  | *Juncus gerardii* | GL | NW Atlantic |
|  | *Juncus inflexus* | GL | Eurasia |
|  | *Najas marina* | GL, SL | Eurasia |
|  | *Najas minor* | GL | Eurasia |
|  | *Pistia stratiotes* | GL | unknown |
|  | *Poa trivalis* | GL, SL | Eurasia |
|  | *Potamogeton crispus* | GL, SL | Eurasia |
|  | *Puccinellia distans* | GL, SL | Eurasia |
|  | *Sparganium glomeratum* | GL, SL | Eurasia |
|  | *Typha angustifolia* | GL, SL | Eurasia |
| **Magnoliopsida** | *Alnus glutinosa* | GL | Eurasia |
|  | *Cabomba caroliniana* | GL | S America |
|  | *Chenopodium glaucum* | GL, SL | Eurasia |
|  | *Cirsium palustre* | GL, SL | Eurasia |
|  | *Conium maculatum* | GL, SL | Eurasia |
|  | *Epilobium hirsutum* | GL, SL | Eurasia |
|  | *Impatiens glandulifera* | GL, SL | Eurasia |
|  | *Lupinus polyphyllus* | GL | N America |
|  | *Lycopus asper* | GL, SL | Mississippi River |
|  | *Lycopus europaeus* | GL, SL | Eurasia |
|  | *Lysimachia nummularia* | GL, SL | Eurasia |
|  | *Lysimachia vulgaris* | GL | Eurasia |
|  | *Lythrum salicaria* | GL, SL | Eurasia |
|  | *Mentha gentilis* | GL, SL | Eurasia |
|  | *Mentha piperita* | GL, SL | Eurasia |
|  | *Mentha spicata* | GL, SL | Eurasia |
|  | *Myosotis scorpioides* | GL, SL | Eurasia |
|  | *Myriophyllum spicatum* | GL, SL | Eurasia |
|  | *Nymphoides peltata* | GL, SL | Eurasia |
|  | *Polygonum persicaria* | GL, SL | Eurasia |
|  | *Rhamnus frangula* | GL, SL | Eurasia |
|  | *Rorippa nasturtium aquaticum* | GL, SL | Eurasia |
|  | *Rorippa sylvestris* | GL, SL | Eurasia |
|  | *Rumex longifolius* | GL, SL | Eurasia |
|  | *Rumex obtusifolius* | GL, SL | Eurasia |
|  | *Salix alba* | GL, SL | Eurasia |
|  | *Salix fragilis* | GL, SL | Eurasia |
|  | *Salix purpurea* | GL, SL | Eurasia |
|  | *Solanum dulcamara* | GL, SL | Eurasia |
|  | *Solidago sempervirens* | GL | NW Atlantic |
|  | *Stellaria aquatica* | GL, SL | Eurasia |
|  | *Trapa natans* | GL, SL | Eurasia |
|  | *Veronica beccabunga* | GL, SL | Eurasia |
| **Spermatopsida** | *Pluchea odorata* var. *purpurescens* | GL | NW Atlantic |
|  | *Pluchea odorata* var. *succulenta* | GL | NW Atlantic |
| **Polypodiopsida** | *Marsilea quadrifolia* | GL | Eurasia |
| **Protozoa** |  |  |  |
| **Euglenida** |  |  |  |
| **Kinetoplastea** | *Trypanosoma acerinae* | GL | Eurasia |
| **Virus** | Rhabdovirus carpio | GL | Eurasia |
|  | Novirhabdovirus sp. genotype | GL | unknown |
|  | IV sublineage b |  |  |
|  | Largemouth Bass Virus | GL | unknown |
